# Combating Root-Knot Nematodes (*Meloidogyne* spp.): From Molecular Mechanisms to Resistant Crops

**DOI:** 10.3390/plants14091321

**Published:** 2025-04-27

**Authors:** Himanshu Yadav, Philip A. Roberts, Damar Lopez-Arredondo

**Affiliations:** 1 Institute of Genomics for Crop Abiotic Stress Tolerance, Department of Plant and Soil Science, Texas Tech University, Lubbock, TX 79409, USA; hiyadav@ttu.edu; 2 Department of Nematology, University of California, Riverside, CA 92521, USA; proberts@ucr.edu

**Keywords:** *Meloidogyne* spp., root-knot nematodes, plant defense system, molecular breeding, omics, genetic engineering

## Abstract

Root-knot nematodes (RKNs; *Meloidogyne* spp.) are significant plant–parasitic nematodes that cause major yield losses worldwide. With growing awareness of the harmful effects of chemical pesticides on human health and the environment, there is an urgent need to develop alternative strategies for controlling RKN in agricultural fields. In recent years, implementing multiple approaches based on transcriptomics, genomics, and genome engineering, including modern platforms like CRISPR/Cas9, along with traditional genetic mapping, has led to great advances in understanding the plant–RKN interactions and the underlying molecular mechanisms of plant RKN resistance. In this literature review, we synthesize the contributions of relevant studies in this field and discuss key findings. This includes, for instance, transcriptomics studies that helped expand our understanding of plant RKN-resistance mechanisms, the overexpression of plant hormone-related genes, and the silencing of susceptibility genes that lead to plant RKN resistance. This review was conducted by searching scientific sources, including PubMed and Google Scholar, for relevant publications and filtering them using keywords such as RKN–plant defense mechanisms, host–plant resistance against RKN, and genetic mapping for RKN. This knowledge can be leveraged to accelerate the development of RKN-resistant plants and substantially improve RKN management in economically important crops.

## 1. Introduction

In recent decades, a sharp rise in the world’s population has created a growing issue of food productivity and human food supply. Nematodes are polyphagous plant parasites that pose a substantial risk to global food security. With the rise in the global temperature, the population of nematodes is impacted, as it alters their life cycle by raising soil temperatures or altering the physiology of host plants, which facilitates nematode reproduction and, ultimately, the infestation process [1]. Nematodes are multicellular animals considered the second most diverse animal lineage after insects. They are members of the phylum Nematoda and have been around for approximately a billion years. They can be found in nature as free-living forms in soil, freshwater, and marine environments, as well as parasites of plants and animals. The three most significant plant parasitic nematode groups are root-knot, cyst, and lesion nematodes. These nematodes are responsible for infecting, feeding, and reproducing on a wide variety of plants, severely limiting crop productivity. In particular, the root-knot nematodes (RKN), *Meloidogyne* spp., exhibit a broad host range, threatening more than 2000 plants, including both monocotyledon and dicotyledon species. Among the more than 90 RKN species, the four most prominent species are *M. incognita*, *M. arenaria*, *M. javanica*, and *M. hapla*, which cause estimated yield losses of 12%, worth $100 billion worldwide annually, and thus, are classified as the most damaging plant pathogens [2]. The symptoms caused by these pathogens involve wilting, stunted growth, leaf discoloration, and deformation of roots.

Global interest in comprehending the relationship between RKN and their host plants is growing due to this severe loss in food productivity [3]. Biological control, non-host crop rotation, and soil additives have been used as control tactics, although these methods offer little protection to plants from RKN infection [4]. Moreover, the extensive use of chemical nematicides is now limited due to the risks they pose to human health and the environment, and the capacity of the nematodes to develop resistance to these chemicals due to repeated application [5]. Host resistance has emerged as a safe and economically viable strategy for controlling RKN in the field, thanks to genetic resistance in the host plants, especially when integrated with other tactics into a management program [6]. However, the availability of commercially viable RKN-resistant crop cultivars is limited for many crops.

To address these challenges, it is crucial to investigate the molecular determinants of plant RKN-resistance mechanisms that can effectively help understand and transfer RKN resistance to economically significant crops. In this literature review, we summarize current knowledge of the molecular and genetic basis of RKN resistance in plants. We integrate and synthesize all relevant literature on plant–RKN interactions, highlighting valuable insights generated through various strategies that researchers employ, including omics approaches, biotechnological strategies, and breeding efforts. We discuss key gaps and future opportunities for developing RKN-resistant crop cultivars. By integrating these insights, we aim to provide a comprehensive review that facilitates an understanding of the mechanisms behind RKN resistance and supports the development of improved crops as an effective and sustainable strategy for managing RKN infestations and safeguarding global crop productivity.

This review was conducted between 2024 and 2025 by searching all bibliographies on plant–RKN interactions in PubMed and Google Scholar databases and following the general recommendations of the PRISMA methodology (https://www.prisma-statement.org/prisma-2020-flow-diagram, accessed on 1 January 2024) for relevant publications. The search was filtered using keywords, including RKN–life cycle, RKN–plant defense mechanisms, host–plant resistance against RKN, genetic mapping for RKN in plants, transcriptomics efforts RKN–plants, and genome engineering/CRISPR/Cas RKN. When the literature regarding these keywords was found, the papers that were most complete and relevant to the study were selected. When possible, the papers with the most citations were included, although relevant papers published fairly recently, with no citations, were also considered due to their contributions being relevant to the field.

To provide some context for the reader, we first present a summary of the establishment of RKN infection in the plant and commonly used practices to control RKN in the field. We then organize the relevant literature into three sections: (i) omics studies to expand our knowledge of the molecular mechanisms behind RKN resistance and guide crop improvement; (ii) genome engineering strategies, including traditional gene overexpression/mutation/silencing and gene editing, to assess gene function and resistance gaining; and (iii) traditional breeding efforts based on molecular markers and associated approaches. Key studies and findings are described and discussed.

## 2. RKN Infection Establishment: Hijacking the Plant Defense System

**Figure 1:**
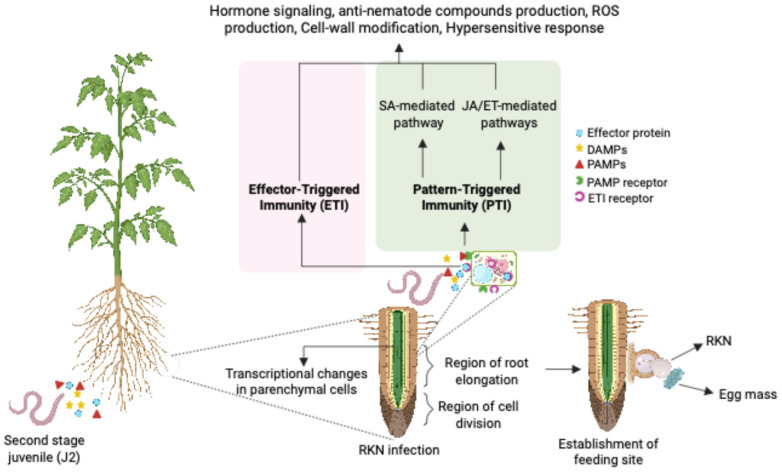
A diagrammatic illustration of RKN (*Meloidogyne* spp.) infection and how plant immunity is triggered. Second-stage juveniles (J2) of RKN infest the plant root’s elongation area, causing transcriptional changes in the parenchyma cells that enable the RKN feeding site to be properly established. The plants’ pattern recognition receptors (PRRs) and effector-triggered immunity (ETI) receptors, respectively, recognize the pathogen-associated molecular patterns (PAMPs) and effector molecules upon infection. This triggers a series of ETI and pattern-triggered immunity (PTI) reactions, which in turn trigger a series of hormonal signals [i.e., salicylic acid (SA), ethylene (ET), jasmonic acid (JA)], and other defense mechanisms to combat the RKN infection. Damage-associated molecular patterns (DAMPs) are released from damaged cells undergoing pathogen invasion [8]. This figure was designed using BioRender (https://www.biorender.com/ accessed on 26 March 2025).

The infection life cycle of RKN begins with the migratory second-stage juveniles (J2) in the soil rhizosphere. Further establishment of the feeding site involves penetration through the root epidermis at the zone of root elongation, moving beyond the Casparian strip to the root tip, and then returning to the vascular cylinder of the roots in search of potential feeding sites. Effectors secreted by the esophageal glands through the RKN stylet play a crucial role in modulating the transcriptional programming of plant parenchymal cells, further leading to the development of giant cells from the feeding site (Figure 1). The development of giant cells involves sequential mitoses without cytokinesis, resulting in an increased number of nuclei and cell size [7]. Pattern-triggered immunity (PTI) and effector-triggered immunity (ETI) are the two kinds of plant immunity active during RKN infection.

In order to ensure appropriate feeding site establishment, RKN modifies the distinct plant developmental pathways controlled by phytohormones. Furthermore, the invasion of the host roots is completed by the defense pathways regulated by phytohormones being taken over. The complex patterns resulting from this manipulation of both defense and development make it difficult for the host plant to determine the precise function of various phytohormones in the RKN parasitism process (Figure 1) [9]. Auxin is known to play a critical role in controlling plant organogenesis. In *Arabidopsis thaliana* (hereafter *Arabidopsis*), the local accumulation of auxins during RKN infection indicates auxin involvement in the proper establishment of the feeding site [10]. Accordingly, studies with *Arabidopsis pin* (1,3,4) mutants demonstrated the crucial role of auxin transport, distribution, and accumulation in facilitating the infection process. When infected with RKN, these mutants showed less susceptibility to the pathogen [11]. Transcriptome analysis demonstrated the intricate temporal and spatial regulation of the auxin biosynthesis and signaling-related genes at the RKN feeding site, corroborating the idea that auxin pathways are altered during RKN infection [3]. Likewise, although less studied, cytokinins have also been shown to be involved in RKN infection. *Arabidopsis* plants with impaired cytokinin levels showed less susceptibility to RKN parasitism [12]. Numerous studies have demonstrated the involvement of salicylic acid (SA) and jasmonic acid (JA) in biotrophic pathogen interactions with regard to hormone-regulated defense pathways. Since RKN is a biotrophic pathogen, SA is expected to mediate immunity against RKN, though the extent of its function appears to vary based on the stage of parasitism [13]. Although SA facilitates the defense response against RKN [14], sometimes the impact is not evident, as observed in tomato (*Solanum lycopersicum*) [15]. A study on transcriptome analysis suggested that RKN inhibits SA-related defense activity in order to colonize the roots [16].

The JA pathway is involved in providing resistance against necrotrophic pathogens and insects. Some studies have shown the upregulation of JA pathways during plant defense against RKN [17]. However, research using mutant lines with impaired JA biosynthesis and perception produced inconsistent results, indicating that a plant’s susceptibility to RKN may vary depending on the precise mutations of JA-related genes [18,19,20]. Wang, et al. [21], demonstrated that RKN infestation in tomato causes electrical and reactive oxygen species (ROS) signals to be sent systemically from roots to leaves, which increased the amount of JAs in the leaves. Grafting with stem sections of mutants lacking GLUTAMATE RECEPTOR-LIKE 3.5 and RESPIRATORY BURST OXIDASE HOMOLOG 1 decreased JA accumulation in the upper stem and leaves and restricted RKN resistance. Furthermore, only the partial activation of mitogen-activated protein kinases (MPKs) 1/2 in leaves was observed due to the lack of ROS and electrical signal transmission across the system, eliminating RKN resistance. This demonstrates how resistance against RKN is provided by systemic signaling through electrical, ROS, and JA signals. A recent report on tomato revealed a complex synergistic relation between SlVQ15, a valine-glutamine (VQ) motif-containing protein, and the transcription factor SlWRKY30IIc, to regulate defense against RKN in the context of JA signaling [22].

In addition, other phytohormones, such as ethylene (ET), abscisic acid, and gibberellins, have been suggested to be involved in RKN infection. However, more research is needed on the regulation of these hormones during RKN parasitism. For additional information, a recent review by Gheysen and Mitchum [9] provides more detail on the regulation of phytohormones during RKN parasitism.

A deeper understanding of the infection process is being achieved by studying proteins secreted by RKN and their interactions with the host cell [23,24]. By degrading, these effector proteins allow RKN to penetrate plant cell walls, evade host defense surveillance, and establish feeding sites. Several effector proteins secreted by RKN that help suppress the host defense mechanism have been identified. One such effector protein, MiMsp40 (*M. incognita* aesophageal gland cell secretory protrein40), when overexpressed in *Arabidopsis*, suppresses the PTI, resulting in a severe infection and increased nematode susceptibility due to an increase in galls and eggs six weeks after inoculation [25,26]. Additionally, host-derived RNA interference of the Mi8D05 effector protein-encoding gene led to a 90% reduction of the RKN infection rate during the J2 stage in *Arabidopsis* [27]. This demonstrates the importance of Mi8D05 during the J2 RKN early infection stage. MiEFF12, another effector molecule released by the RKN esophageal gland and located in the endoplasmic reticulum, has likewise been shown to modify tomato host immunity by targeting the basic leucine zipper 60 (BZIP60) and plant bap-like (PBL) proteins [28]. Another effector protein called MiPFN3 (*M. incognita* Profilin 3) was found to promote parasitism by directly altering the cytoskeleton of plant cells to cause gall formation. This type of effector is an actin-binding protein, structurally similar to plant profilin. This structural similarity allows MiPFN3 to bind to actin monomers and potentially sequester them, impeding proper actin polymerization. This interference in plant actin dynamics disrupts the plant’s normal cell structure and function, resulting in the development of giant cells that serve as a feeding site for the RKN [29].

Certain effector proteins also target transcription factors (TFs) in plants. For example, the effector protein 16D10 has been shown to interact directly with the SCARECROW-like (SCL) TFs in *Arabidopsis*. This interaction results in an abnormal root growth pattern, facilitating RKN infection [30,31]. Recent studies with the Mi2G02 effector protein have expanded our understanding of how it manipulates GT-3A (Trihelix transcription factor GT-3a) TF to reprogram gene expression, favoring the development of feeding sites in *Arabidopsis*. It targets the host nuclear processes by interacting with the transcriptional machinery. Mi2G02 was found to stabilize GT-3A protein by inhibiting the proteosome-dependent pathway. Further, GT-3A knockout resulted in fewer egg masses, whereas its overexpression increased plant susceptibility to *M. incognita.* Although the structural analysis for Mi2G02 is limited, functional experiments show that it operates as a transcriptional repressor, modulating host gene expression to suppress immune responses and promote a favorable environment for nematode development [32]. Other effector proteins, such as MiSGCR1, MeTCT, and MgGPP, secreted by RKN have been shown to suppress plant cell death caused by the hypersensitive response. These examples show how RKN manipulates host root cells and suppresses plant immunity by using a variety of effector proteins. However, since most effector proteins are not yet fully characterized, there is much more to discover about the effector proteins produced by RKN [33,34,35].

## 3. Common Strategies for Controlling RKN

### 3.1. Cultural Practices and Pesticides

Traditional tactics such as crop rotation, cover cropping, flooding, and solarization have been employed to mitigate RKN infestations [36,37]. However, these methods are often only partially effective due to the wide host range and diversity of RKN species found in the soil. Furthermore, methods such as flooding require warm climates, abundant water, and prolonged application periods, which could be detrimental to crop plants. Similarly, solarization demands a prolonged stoppage of crop cultivation, significant investment, and careful planning.

A proven control method involves fumigating the soil with chemicals with nematicidal activity before planting. Fumigants, including 1,3-Dichloropropene, metam sodium, and methyl bromide, are effective at controlling RKN parasitism in plants [38,39]. However, due to their significant detrimental impact on the environment and public health, these chemicals are increasingly restricted or prohibited in many countries [40]. Alternative approaches, such as soil amendments, plant hormones, and bio-fertilizers have been explored to minimize reliance on hazardous chemicals. For example, when lime and ammonium bicarbonate are combined, ammonia is released, having nematicidal activity [41]. Mustard seed meal has also been used in the field to lower RKN parasitism. Likewise, the application of SA to both the leaves and roots of the plant has been suggested to successfully control RKN [42]. However, in contrast to their counterparts, which have a more robust and wide-ranging impact in suppressing RKN populations, these organic approaches are less successful [43].

### 3.2. Biopesticides

Biopesticides represent another avenue to control RKNs, which are proven to be safe in terms of environmental and public health. These include beneficial microorganisms such as bacteria and fungi, which are ingested by the nematodes, causing tissue destruction or secretion of toxic chemicals that impact the host nematode [44]. Their modes of action vary with the nematode’s developmental stage. For instance, *Paecilomyces lilacinus* targets and invades *M. incognita* eggs in tomato plants [45], while *Arthrobotrys* spp., a saprophytic fungus, captures nematodes using its three-dimensional hyphal network and degrades their cuticle using extracellular enzymes [46]. Entomopathogenic nematodes, such as *Heterorhabditis bacteriophora* and *Steinernema carpocapsae*, have also demonstrated substantial efficacy, reducing RKN populations in fig (*Ficus carica*) plants by 75.4% and 83.4%, respectively [47]. Unfortunately, the practical use of biocontrol agents for nematode control is constrained by environmental factors such as soil composition, plant susceptibility, and microbial compatibility, limiting their widespread adoption [48].

### 3.3. Host Plant Resistance

Host plant resistance has been shown to be an effective, economical, and environmentally safe RKN management strategy. Cultivars are typically classified as resistant or tolerant based on their response to nematode pressure; resistant genotypes limit or prevent RKN reproduction, thereby reducing pathogen load, while tolerant genotypes exhibit minimal damage and may maintain yields despite infection. While tolerance may help sustain productivity, resistance is particularly valuable for long-term nematode control as it helps reduce the pathogen population. In Upland cotton (*Gossypium hirsutum*), for example, extensive screening efforts across wild relatives and cultivated genotypes have uncovered several key resistant sources. Among them, the Auburn 623 RNR line stands out for its near immunity to RKN. The transgressive segregation in the cross between two moderately resistant parents, Clevewilt 6 and Wild Mexican Jones (WMJ), is thought to be the source of Auburn 623 RNR’s near immunity [49,50]. Resistance from Auburn 623 RNR has been successfully introgressed into elite cultivars, including M-120 RNR, M-155 RNR, M-315 RNR, and M-240 RNR, through backcrossing. These M-derived lines combine strong RKN resistance with improved agronomic performance. Additional resistant cultivars include LA434-RKR and its derivatives (Paymaster H1560 and Stoneville LA887) and Acala NemX [51,52]. Pinpointing RKN-resistant genotypes by implementing different approaches, including the use of chromosome substitution lines [53] and understanding the impact of transgressive effects of multiple resistance loci contributed from different parents [54,55], will enable the improvement of agronomically related traits, as demonstrated in cotton [56].

## 4. Omics Approaches to Understanding Plant–RKN Interactions and Resistance

There has been a revolution in our understanding of plant biology due to access to large-scale omics datasets, including genomics, transcriptomics, proteomics, metabolomics, metagenomics, and phenomics. The system-level approach has emerged due to this ground-breaking access to omics data, which offers unprecedented insights into the mechanisms behind diverse and complex biological processes and how plants respond to biotic and abiotic stresses, including interactions with RKN. By enabling rapid and cost-effective data generation that is useful for crop improvement, compared to traditional breeding methods, omics technologies have both expanded research capabilities and introduced new analytical challenges (Table 1).

The advancement of next-generation sequencing (NGS) technologies and subsequent improvements in genomic data analysis have led to high-throughput data generation for genomes [single nucleotide polymorphisms (SNPs), loss of heterozygosity variants, copy number variants (CNVs), genomic rearrangements, and rare variants], transcriptomes (differential expression of genes, alternative splicing, small RNAs like miRNAs, and long non-coding RNAs), and epigenomes (DNA methylation, histone modification, chromatin accessibility, and transcription factor binding) [59]. 

Five major steps—sample collection, high-quality nucleic acid extraction, library preparation, clonal amplification, and sequencing, which can be accomplished through diverse platforms—form the foundation for both genomics and transcriptomics-based data generation. Furthermore, depending on the intended downstream application, specific approaches are employed for each step. Sequencing is followed by data cleaning, filtering, assembly alignment, variant calling, variant annotation, and ultimately, functional prediction. Heterogeneous datasets present difficulties because different individual datasets require distinct quality assurance, quality control, data normalization, and data reduction strategies. For instance, the normalization of bulk RNA-sequencing (RNA-seq) data differs from that of small RNA-seq data, as RNA-seq datasets comprise tens of thousands of transcripts, while small RNA-seq datasets typically include fewer than 2000 small RNAs.

Interpreting genome and transcriptome data in the context of biological function, which entails the influence of specific variants on phenotypic variation, presents additional challenges [60]. To bridge the genotype–phenotype gap, researchers increasingly integrate metabolomic and proteomic data with genomic and transcriptomic datasets since it provides the molecular and metabolite information that connects genetic and epigenetic variations with the phenotypic factors [61]. Multi-omics integration, especially combining transcriptomics, proteomics, and metabolomics, holds great potential for understanding complex traits like RKN resistance. However, it has several limitations. A notable issue is the discrepancy between transcriptomic and proteomic data, as mRNA abundance does not necessarily correlate with protein levels due to a variety of factors, such as post-transcriptional regulation, translational efficiency, and protein degradation. This often complicates a straightforward functional interpretation solely from RNA-seq results. Weighted Gene Co-expression Network Analysis (WGCNA) helps address these limitations. WGCNA is used to build gene-co-expression networks from RNA-seq data, allowing the identification of gene modules associated with a specific trait or condition. These modules can be functionally annotated or combined with proteomics and metabolomics data to provide a deeper understanding of the regulatory networks that underpin traits such as RKN resistance. Despite its promise, multi-omics data integration remains challenging due to the lack of standardized pipelines, differences in data scale and format, and limitations in computational resources [62,63,64]. Among the omics approaches, genomics and transcriptomics are currently the most mature in terms of available laboratory reagents, standardized protocols, analytical tools, and public data repositories. They offer valuable opportunities to obtain high-quality data from small amounts of tissue to address a wide range of biological questions. Numerous studies have leveraged these technologies to investigate RKN parasitism in key crop species, yielding high-quality datasets from minimal tissue input. Long-read sequencing platforms such as Pacific Biosciences (PacBio) and Oxford Nanopore Technologies (ONT) provide unparalleled advantages over short-read platforms, allowing read lengths typically ranging from 10 to 100 Kb, and in some cases, ultra-long reads (2.3–4 Mb). These extended reads facilitate the detection of complex structural variants and improve genome assembly, particularly in repetitive regions.

Recent advances in single-cell (sc) multi-omics technologies now enable simultaneous profiling of the transcriptome, epigenome, and chromatin architecture at single-cell resolution [65]. This approach facilitates a more comprehensive examination of the complex molecular mechanisms that regulate gene expression and cellular heterogeneity, providing a more accurate depiction of individual cell states. Sc-multi-omics is particularly well-suited for applications involving rare cell types, as it maximizes the information obtained from each individual cell. This technology is promising for studying how the transcriptome profile changes at the single-cell level in plant roots in response to RKN infection, which could lead to new avenues for integrating molecular resistance to RKN in different crops and identifying cell-specific determinants related to infection and resistance mechanisms.

There are challenges associated with sc-multiomics, such as data sparsity and noise, gene/allelic dropout, high sequencing costs, and low recovery efficiency per cell. Sequencing costs create a constant trade-off between throughput and the richness of information gathered. This results in limited coverage per individual cell, contributing to data sparsity at various levels. While single-cell omics hold great promise for revealing root cell-type-specific responses to RKN infection, research in this area remains in its infancy and warrants further exploration. Due to the demonstrated relevance of transcriptomics approaches to identify mechanisms and candidate genes that can subsequently be assessed to confer stress resistance, we discuss relevant studies in this field below.

### Transcriptomic Efforts Toward Understanding Plant Resistance to RKN

Transcriptomic profiling has become an essential tool for dissecting plant immune responses during plant–pathogen interactions. This approach uses high-throughput sequencing platforms to generate pair-end reads, allowing comparative analysis of transcriptomic changes between resistant and susceptible genotypes in response to RKN infection [66]. By identifying differentially expressed genes (DEGs), it provides insights into regulatory and metabolic pathways that are activated or suppressed during infection, and enables the annotation of novel transcribed regions, alternative splice variants, and genetic polymorphism [67]. Identified DEGs potentially hold the key to understanding the resistance mechanism.

In *Arabidopsis*, transcriptomic analysis of gall formation during RKN infection revealed 3373 DEGs associated with diverse biochemical and physiological functions, including metabolism, energy production, development, aquaporins, and cell cycle-related processes [68]. Similarly, in African rice (*Oryza glaberrima*), RNA-seq with histological analyses demonstrated that the resistant variety TOG5681 activates defense responses involving JA, SA, and ET signaling pathways, and pathogenesis-related (PR) proteins and phenylpropanoids. Key genes such as *OsLOX7* (lipoxygenase 7), *OsAOS2* (allene oxide synthase 2), *PAL* (phenylalanine ammonia-lyase)*,* and *OsThion2* (thionin2) exhibited high basal expression, contributing to reduced RKN penetration, impaired feeding site development, and limited reproduction. These responses likely promote a localized hypersensitive response, conferring resistance to RKN [69].

Comparative transcriptomic analysis between RKN-susceptible and resistant genotypes has provided valuable insights into the RKN infection process and plant resistance mechanisms. In cotton, a transcriptomic analysis comparing the susceptible Acala SJ2 with resistant cultivars Acala NemX and WMJJ revealed that resistant lines exhibit constitutive expression of defense-related genes, even in the absence of infection. Notably, JA and SA pathway genes such as *NPR1/3* and *COI-JAZ*, as well as TIR-Nucleotide Binding Site-LRR proteins (TIR-NBS-LRR) and PAMP-RLKs receptors, were up-regulated in resistant NemX. Two candidate genes, orthologs of the *Arabidopsis TIR-NBS-LRR* gene (*AT5G36930*), were located within a known QTL in chromosome A11 linked to RKN resistance, highlighting potential targets for marker-assisted selection [70]. A time-course RNA-seq analysis across five developmental stages after RKN infection with the cotton varieties Cocker 201 (susceptible) and M-120 RNR (resistant) also revealed a rich collection of pathogenesis-related (PR) genes, ligands, and receptors as potential candidate genes [71].

A comparable transcriptomic strategy has been applied to cowpea (*Vigna unguiculata*), focusing on the *Rk* resistance locus [72]. Resistance to *M. incognita* and *M. javanica* has been mapped using extensive phenotyping under field and controlled conditions, enabling the identification of resistance QTLs [73,74]. Integration of transcriptomic and histological analyses in near-isogenic cowpea genotypes has further characterized gene expression at nematode feeding sites, aiding functional validation of candidate genes [75,76]. A recent report on the comparative RNA-seq analysis between the tomato cultivars SL-120 (resistant) and GAT-5 (susceptible) revealed that the *Mi-1* gene, widely known to confer RKN resistance in diverse species, presents root-specific expression in the resistant genotype and that it exerts this effect by inducing resistance to programmed cell death, plant defense, and cellular remodeling, leading to lignin deposition against the pathogen [77].

Studies in additional crops, including alfalfa (*Medicago sativa*), eggplant (*Solanum melongena*), tobacco (*Nicotiana tabacum*), pepper (*Capsicum annuum*), sweetpotato (*Ipomoea batatas*), and soybean (*Glycine max*), consistently report transcriptional activation of defense signaling, hormone pathways (especially ET), and immune receptors in RKN-resistant cultivars (Table 2). Collectively, transcriptome profiling provides foundational knowledge of plant defense regulation during RKN infection and informs resistance breeding strategies. These efforts can be further strengthened using single-cell transcriptomics to further resolve the spatial dynamics of plant responses. In *Arabidopsis*, gene co-expression network analysis under bacterial and fungal infection has revealed gene modules enriched for pathogen-responsive genes, including PR-encoding proteins, and plant hormone biosynthesis and signaling components [78]. Similar studies focusing on RKN could identify key regulatory nodes and refine our understanding of how resistance is orchestrated even at the cellular level.

## 5. Genome Engineering Approaches to Improve RKN Resistance

**Figure 2:**
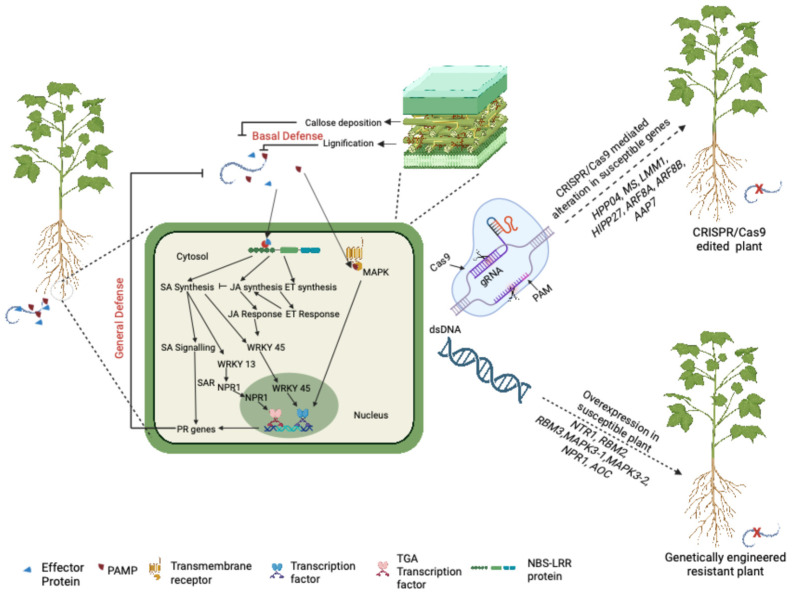
A diagrammatic representation of the defense response of plants to RKN (*Meloidogyne* spp.) infection and the elements that contribute to plant resistance that may be utilized to develop RKN resistance in susceptible genotypes. The cell wall’s cellulose and lignin deposition is part of the basal defense. In general, plant defense response, PRRs detect PAMPs, whereas effector proteins are identified by nucleotide-binding leucine-rich repeat (NLR) proteins. Upon detection, the plant initiates an array of defense signaling pathways, including hormone regulation and mitogen-activated protein kinase (MAPK) cascades. Upon activation, transcription factors such as WRKY upregulate SA signaling, which further upregulates PR genes, strengthening the plant’s defense response and limiting RKN infection. Similarly, MAPK signaling enhances transcriptional control of PR genes, hence increasing resistance to RKNs. In another scenario, CRISPR/Cas9 could be utilized to alter the s-genes expression, thus enabling the plants to attain resistance against RKN. SA: salicylic acid; JA: jasmonic acid; ET: ethylene; PR: pathogenesis-related genes; PRR: pathogen recognition receptors; PAMP: pathogen-associated molecular pattern; gRNA: single guide RNA; PAM: protospacer adjacent motif; dsDNA; double-stranded DNA [87,88,89,90]. This figure was designed using BioRender (https://www.biorender.com/ accessed on 26 March 2025).

Recent advances in genome engineering approaches are opening up a new era for accelerating the development of crops resistant to biotic and abiotic stresses. Due to continuous research and progress in biotechnology, it is now possible to incorporate and express genes from one organism into another. Genome engineering has led to the improvement of both quality and quantity in terms of crop yield and production. Several crops have been modified using genetic engineering approaches to make them resistant to insects [e.g., *Bt* cotton, *Bt* maize (*Zea mays*)], herbicides (roundup-ready soybean, cotton, maize), and rust (wheat, *Triticum aestivum*), which are now commercially available. Genome engineering approaches have been employed to impart RKN resistance to monocotyledon and dicotyledon plant species, including important crops such as tomato, soybean, cotton, and eggplant. However, these are only proof-of-concept studies thus far, and engineered plants are generally tested only under greenhouse conditions. The approaches that could be utilized to generate RKN-resistant plants are shown in Figure 2. To the best of our knowledge, no RKN-resistant engineered crops are commercially available yet.

### 5.1. Overexpression, Silencing, and Mutation of Genes to Develop Resistance to RKN

Heterologous expression of *R* genes has been an attractive option for conferring resistance against RKN in plants. *Mi-1* was the first *R* gene with NBS-LRR domain isolated from tomato and is known to provide race-specific RKN resistance [77,91]. Since then, several *R* genes have been isolated and cloned to provide resistance in different crops. For example, Zhang, et al. [92] validated the functional role of the CC-NB-LRR domain-containing *GhNTR1* gene in tobacco, which conferred RKN resistance by inducing a hypersensitive response and, eventually, cell death. This study revealed the involvement of peroxidase-targeting miRNA (miR413) in the RKN resistance mechanism, thus providing insights into how miRNAs may govern RKN resistance. Similarly, two TIR-NB-LRR domain-containing *R* genes (*RBM2* and *RBM3*), which provide resistance to RKN, were isolated from *Prunus sogdiana* based on NB-ARC domain similarity with previous *R* genes. This indicates the relevance of identifying and cloning novel *R* genes with conserved NB-ARC domains in crops where genomic data are still poor [93,94]. Interestingly, when expressed in tobacco, *PsoRBM2* was shown to interact with the heat shock chaperone complex (PsoRPM2-HSP90-1-SGT1-RAR1), thus enabling the tobacco plants to develop resistance against RKN. On the other hand, *PsoRBM3* was found to positively regulate hormonal signaling (JA, SA, and ET) pathway genes to confer RKN resistance [94]. This suggests the concerted action of different molecular mechanisms orchestrated by these *R* genes to provide RKN resistance. It will be interesting to investigate whether *PsoRBM3* is also working in cooperation with the chaperone complex.

Though the heterologous expression of *R* genes has been shown to be an effective strategy to develop RKN resistance, there have been some cases where this approach with *R* genes did not result in the desired resistance response. For instance, when the *Mi1.2* gene from tomato was heterologously overexpressed in eggplant, the resulting engineered plants successfully showed the RKN-resistance phenotype as expected [95]. However, when the same gene was transferred to tobacco and *Arabidopsis*, it did not confer any resistance to the RKN [96]. These results indicate that the *R* genes are crop-specific and the molecular mechanisms providing pathogen resistance are crop-relative. This challenge could be potentially overcome if the *R* genes are sourced from closely related plant species.

PTI and ETI are both mediated by MAPKs. Thus, heterologous expression of *MAPK* genes in different crops would also assist in developing RKN resistance in susceptible crops, as achieved in Upland cotton. The overexpression of two soybean genes, *GmMAPK3-1* and *GmMAPK3-2*, separately resulted in the impairment of RKN development and reproduction [88]. Overexpression of *MAPK3-1* led to reduced root galls, egg masses, and invasive J2 juveniles by 80.32%, 82.37%, and 88.21%, respectively. However, there was an unexpected increase in the egg number by 28.99%, but the J2 nematodes were inviable. Similar results in terms of reduction in root galls, egg masses, and J2 nematodes were obtained by overexpressing *MAPk3-2*. Furthermore, the reproductive factors of RKN in both the overexpressing lines *MAPK3-1* and *MAPK3-2* were reduced by 60.39% and 46%, respectively [88].

Transgenic plants expressing genes involved in plant hormone signaling pathways have been shown to exhibit increased resistance to RKN in several plant species. This was shown for the heterologous expression of *NPR1* (*non-expressor of pathogenesis-related genes-1*), encoding a transcriptional activator involved in the SA signaling pathway and known to induce SAR, driven by the expression of secretory protein genes (*PR-1* and *PR-5*). The expression of *Arabidopsis* and soybean *AtNPR1* and *GmNPR1* in tobacco and Upland cotton, respectively, resulted in a significant reduction in the formation of root galls and egg masses in transgenic plants in a dose-dependent manner in terms of *NPR1* expression [97,98]. A study conducted by Fan and co-workers [19] revealed the importance of JA signaling in suppressing RKN infection in tomato. A comparison of the JA biosynthetic mutant, *spr2 (suppressor of prosystemin-mediated responses2)*, and the CaMV35S::*prosystemin* overexpressing lines showed differences in JA levels in these two lines. The *spr2* mutant lines showed 10% less JA content and more susceptibility to RKN. In contrast, the CaMV35S::*prosystemin* lines were more resistant due to up-regulated expression of *proteinase inhibitor II* (*PI-II*) upon RKN infection, as PI-II level was increased in CaMV35S::*prosystemin* lines. Exogenous foliar application of JA in *spr2* mutant lines induced a regain of resistance by up-regulating *PLA_2_* (*phospholipase A_2_*) and *PI-II* expression, thus indicating the significant role of methyl jasmonate (MeJA) in mitigating RKN susceptibility. Recently, the RKN resistance gene (*NtRK1*) was cloned from the tobacco variety TI706 and overexpressed in the susceptible variety Changbohuang. This led to increased tolerance to RKN infection by upregulating phytohormonal signaling, specifically JA and SA. Meanwhile, the RNAi lines for *NtRK1* of the resistant variety K326 showed decreased resistance after RKN infection, indicating that *NtRK1* provides RKN resistance by coordinating JA and SA signaling [99]. Interestingly, *NtRK1* shows high homology with genes *CaMi* in pepper, and *Mi-1.1* and *Mi-1.2* in tomato, which confer RKN resistance.

Independent studies in tomato validated the role of two WRKY transcription factors, *SIWRKY3* and *SIWRKY45,* in regulating hormonal signaling during RKN infection. These studies concluded that *SIWRKY3* is a positive regulator of SA and Auxin (IBA) signaling, along with the accumulation of related molecules from oxylipin and shikimate pathways upon RKN infection. On the other hand, *SIWRKY45* acts as a repressor of JA signaling by binding to the promoter of the *ALLENE OXIDE CYCLASE* (*AOC*) gene of the JA pathway, thus, inhibiting its expression [89,100]. Two cotton genes, *GhDIR4* (*dirigent protein4*) and *GhPRXIIB* (*peroxiredoxin 11B*)*,* were expressed in *Arabidopsis* and showed significant improvement in resistance to *M. incognita* by impairing the female maturation process [101]. Similarly, based on expressed sequence tag sequencing and previous studies, the gene candidate DUF538 (domain of unknown function538) with unknown functional annotation was tested in peanut, soybean, and *Arabidopsis* and found to impart RKN resistance. DUF538 was shown to up-regulate genes involved in JA and ET signaling pathways and redox signaling [102]. However, the complete knowledge of how it provides resistance remains unclear.

Expressing anti-nematode proteins or proteases is another promising approach to generating RKN-resistant crop cultivars, and several efforts have been made in this field. For example, cystatin (cysteine protease inhibitor), a well-known protease, has been expressed in eggplant, potato (*Solanum tuberosum*), tomato, and rice (*Oryza sativa*) to impart RKN resistance [103,104,105,106]. The main function of cystatin is to inhibit the exogenous proteins encoded by the pathogen, thus inhibiting their growth. An important consideration is using suitable promoter sequences to express those proteases, as reducing exposure to non-pathogenic organisms is critical. For instance, tissue-specific promoters like TUB-1, which is a root-specific promoter, would offer a better option [106]. A different anti-nematode protein, Bt (*Bacillus thuringiensis*) crystal protein Cry5Ba2, has been effectively overexpressed in tomato and tobacco root leucoplasts and exhibits strong resistance to RKN [107]. A new understanding of RKN feeding behavior and their capacity to consume leucoplast protein is provided by the study, which shows that the female RKNs ingest the nematicidal protein through plastids rather than the cytosol.

### 5.2. Genome Editing for Understanding and Developing Resistance to RKN

With the development of genome editing tools, particularly CRISPR/Cas9 (clustered regularly interspaced short palindromic repeats/CRISPR-associated protein 9), it is now feasible to edit specific genes to introduce desired traits, such as disease resistance, enhanced nutrient uptake, enhanced nutritional value, among others, thereby generating transgene-free plants. To date, CRISPR/Cas9 has been used to create a variety of crops [e.g., rice, barley (*Hordeum vulgare*), tomato, and wheat] resistant to various biotic stressors (bacterial, fungal, and viral); for more details, readers are referred to this reference [108].

To the best of our knowledge, only a few studies have been carried out that use CRISPR/Cas9 to develop resistance to RKN by targeting the Susceptibility (*S*) genes. The S genes are plant genes that are induced or targeted by pathogens to recognize the host, and for penetration, nutrient uptake, proliferation/spreading, and suppression of the host immune system [109]. Huang, et al. [110] targeted the S gene *OsHPP04* (*copper metallochaperone heavy metal-associated plant protein 04*), a negative regulator of plant host immunity, using the CRISPR/Cas9 system to knockdown gene function in rice. Compared to the wild type, the *OsHPP04* CRISPR/Cas9-driven mutant lines displayed increased levels of ROS, callose deposition, and the expression of genes involved in defense. The homozygous transgene-free lines displayed enhanced tolerance to RKN without impairing plant development. In soybean, malectin-like receptor kinase (GmLMM1) modulates cell death and pattern-triggered immunity during RKN infection. RKN encodes rapid alkalinization factor (RALF)-like ligands, which bind to GmLMM1 and suppress the host immune response, hence increasing RKN infection. Derived from investigations with ethyl methanesulfonate (EMS) soybean mutants, the CRISPR/Cas9 system was utilized to mutate *GmLMM1*, demonstrating that *GmLMM1* is a negative regulator of RKN resistance [111]. One of the edited lines showed a combination of resistance and tolerance to RKN, displaying a lower number of nematodes than the wild type during early infection and full repression of gall formation 30 days after infection.

Using hairy root transformation in cucumber (*Cucumis sativus*), the malate synthase (*CsMS*) gene, which is involved in malic acid synthesis in the glyoxylate cycle, was knocked out using CRISPR/Cas9. The CRISPR/Cas9 mutant lines of the *CsMS* gene have been demonstrated to reduce the number of RKN females and eggs, as well as the gall count and giant cell size. This could be attributed to reduced root metabolic activity caused by the lack of function of the *CsMS* gene [112]. In tomato, two auxin-responsive transcription factors, *SlARF8A* and *SlARF8B*, were identified as susceptible factors that promote giant cell development after RKN infection. The *SlARF8A* and *SlARF8B* knockout lines showed a 50% reduction in gall number and egg mass compared to the wild type, as well as a 30% reduction in the size of large cells upon RKN infection [113]. In *Arabidopsis*, two S genes, *AtHIPP27* and *AtAAP6*, were knocked out using the CRISPR/Cas9 approach, and the homozygous transgene-free lines were subsequently challenged with RKN. The mutant lines exhibited higher RKN resistance as compared to the wild type, as RKN multiplication was reduced by 64.87% and 56.28%, respectively [90,114].

These studies have demonstrated the potential of CRISPR/Cas9-mediated editing of *S* genes to generate RKN-tolerant or resistant lines, offering a novel strategy for nematode management. Targeting specific *S* genes using CRISPR/Cas9 not only enables the development of resistant cultivars but also provides valuable insights into plant–nematode interactions. Expanding this approach to other economically important RKN-susceptible crops could be highly beneficial. However, the success of such applications depends on the accurate identification and thorough characterization of key *S* genes, which would maximize the precision and utility of CRISPR/Cas9-based interventions.

Despite its promise as a powerful tool for targeted genome editing, the application of CRISPR/Cas9 is not without limitations. One major concern is the risk of off-target mutations, particularly in genomic regions where the target gene shares high sequence similarity with other genes or is embedded in complex chromatin structures [115]. Addressing these challenges through improved target design and off-target detection strategies will be crucial for translating molecular innovations into robust, field-deployable RKN resistance in crops.

## 6. Genetic Approaches to Combat RKN Infestation

The successful improvement of RKN resistance through conventional breeding approaches relies on the presence of resistance alleles within the gene pool of the target crop. Tomato RKN resistance is one prominent example, where resistance to RKN is conferred by the *Mi* gene, originally derived from wild tomatoes. Hybridization with these wild tomato plants has led to the development of all modern commercial tomato cultivars carrying *Mi*-mediated resistance [116].

**Figure 3:**
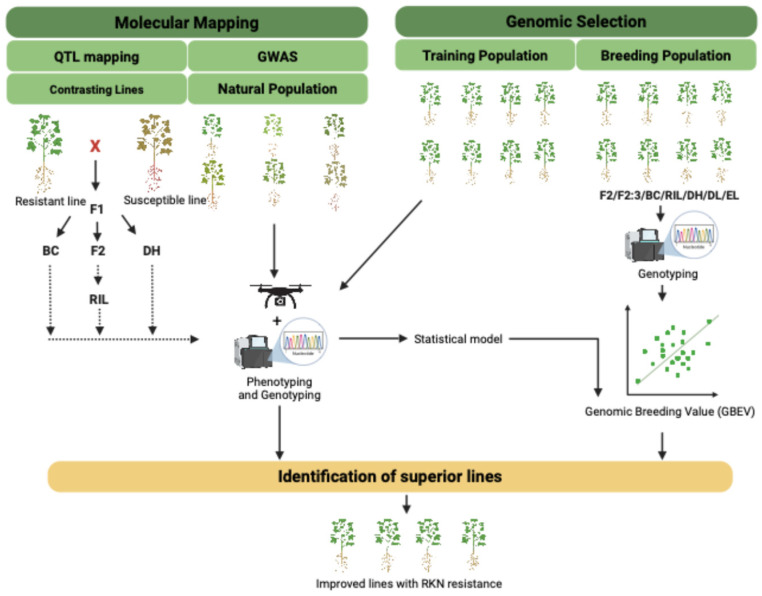
Flowchart representing the integrated approaches used to identify superior RKN (*Meloidogyne* spp.)-resistant genotypes. QTL mapping and GWAS require genotyping and phenotyping data, which can also be used as training sets to carry out genomic selection effectively. Finding the superior lines with RKN resistance can be accomplished through the integration of these methods [61,120]. This figure was designed using BioRender (https://www.biorender.com/ accessed on 26 March 2025).

Typically, classical molecular genetics is commonly used to identify genes underlying discrete traits, while more complex traits are dissected using QTL mapping [117]. Modern breeding techniques integrate these molecular techniques with traditional methods to enhance genetic gain (Figure 3). This strategy aims to link genotype and phenotype by using genomic and molecular tools to improve desirable crop traits [118]. Advances in bioinformatics, statistical methodologies, and the increase of molecular databases, supported by high-quality reference genomes, now facilitate a deeper understanding of plant–RKN interactions at the molecular level. Tools such as marker-assisted selection (MAS), genotyping-by-sequencing (GBS), genome-wide association mapping (GWAS), and genomic selection (GS), are accelerating efforts to breed RKN-resistant cultivars [116,119]. These integrated approaches offer a comprehensive framework for RKN management and hold promise for sustainable crop protection. The following sections explore these strategies in more detail.

### 6.1. Marker Assisted Selection

In MAS, the linked markers’ binding patterns are used to indirectly select the desired plant phenotype. MAS is based on the premise that tightly linked markers can reliably indicate the presence of a target gene. Because molecular markers serve as chromosome landmarks, they are invaluable tools for dissecting plant–nematode interactions and facilitating the introgression of resistance genes associated with agronomically important traits.

MAS has been effectively used to combine and stack resistance genes in several crops, including wheat [121], rice [122], and soybean [108], mirroring its successful application in tomato cultivars to pyramid numerous disease resistance traits [123]. Compared to other breeding practices, MAS has both pros and cons. Firstly, it is more cost-effective, does not involve hazardous chemicals or specialized tools, and can be performed in greenhouse and field tests under specific nematode pressure. However, the process can be time-intensive, particularly when identifying molecular markers closely linked to the resistance genes and incorporating these genes into susceptible cultivars. Nevertheless, molecular markers have become essential tools in breeding programs for economically important traits, making MAS a preferred approach in many crops.

Various approaches based on molecular markers such as cleaved amplified polymorphic sequence (CAPS), sequence characterized amplified region (SCAR), amplified fragment length polymorphisms (AFLPs), restriction amplified length polymorphisms (RALPs), random amplified polymorphic DNA (RAPD), SNPs, and reverse-transcription polymerase chain reaction (RT-PCR), have been used to identify cultivars with resistance to the RKN [124,125]. In tomato, for instance, molecular markers linked to the *Mi-1* gene enabled the rapid identification of the resistance alleles without requiring nematode inoculation. *Mi-1* homologs confer resistance against a wide range of pests and pathogens, including the most common RKNs, i.e., *M. javanica*, *M. incognita*, and *M. arenaria* [116,126]. To find potential breeding lines and quickly test germplasm for nematode resistance, a combination of conventional screening techniques and molecular markers is applied. Among the markers used, the SCAR marker Mi23 has proven more reliable than REX-1, which can yield false positives for *the Mi-1* gene [127]. Further analysis of the *Mi-1* locus has revealed several *Mi-1* homologs in that chromosome region, some of which do not confer resistance to RKN, while at least one conferred heat-stable RKN resistance [128].

In cotton, significant efforts have been made to identify molecular markers closely linked to the southern RKN-resistance genes. Several studies have shown that resistance to RKN is associated with SSR markers on chromosomes 11 and 14 [129]. Additionally, to facilitate its application for RKN resistance, an AFLP marker was developed into a CAPS marker, which is a single-locus PCR marker, as AFLPs are redundant in nature and less suitable for selection purposes [130]. Using recombinant inbred lines (RIL) of cotton, two single sequence repeats (SSR) markers, namely CIR 316-201 and BNL 3661-185, were confirmed to be linked to RKN resistance on chromosomes 11 and 14 [131]. Their effectiveness was further validated in MAS using two RKN-resistant crosses, namely M240RNR × FM966 and Clevewilt 6 × Mexico Wild (PI563649). Subsequently, the F_2_ populations were phenotyped for gall index and RKN egg number per plant and genotyped for CIR 316 (Chr 11) and BNL 3661 (Chr 14). It was concluded that the MAS was effective, and the identified markers on Chr 14 were mainly linked with a dominant RKN resistance gene, which affects RKN reproduction [132]. Another study identified three additional SSR markers (BNL 3279_114, BNL 1066_156, and BNL 836_215) on chromosome 11 linked to RKN resistance in cotton [125]. Molecular markers for RKN resistance breeding for other important crops are summarized in Table 3.

### 6.2. QTL Mapping Efforts for RKN-Related Traits

QTL mapping is a widely used approach for investigating genetic architecture and DNA marker associations in segregating biparental populations. By analyzing trait-marker associations and marker–marker interactions, it is possible to identify QTLs on a genetic linkage map. Numerous QTLs related to RKN have been identified and genetically mapped in several crops (Table 4).

In cotton, studies using recombinant inbreeding transgressive segregating lines derived from Texas Marker-1 (TM-1, *G. hirsutum*) and Pima 3-79 (*Gossypium barbadense*) revealed four major QTLs linked to RKN resistance located on chromosomes 3, 4, 11, and 17. These QTLs account for 8.0–12.3% of the phenotypic variance in root galling. Additional QTLs on chromosomes 14 and 23 were linked to variation in the number of eggs per gram of root tissue, collectively accounting for 9.7% to 10.6% of the variation [146]. The Mi-C11 QTL on chromosome 11, identified in the resistant M-120 RNR line [147], was further validated in RKN-resistant Upland cotton lines, Acala NemX, and Pima S-7, confirming its role in RKN resistance. This locus was determined to contain multiple resistance genes, including *rkn1* and a transgressive factor, *RKN2* [54,148]. Functional analysis of *MIC-3* (*Meloidogyne Induced Cotton*) by Wubben, et al. [149], previously mapped on chromosome 14 in resistant cotton, revealed epistatic interactions with the chromosome 11 QTL, suggesting distinct contributions to gall formation and nematode reproduction. Additional reports have also confirmed the significance of known QTLs located on chromosomes 11 and 14, which confer resistance against RKN in cotton [150].

Beyond cotton, QTLs associated with RKN resistance have been reported in carrot [151,152], maize [153], sweetpotato [154], peanut [155], cucumber [156], sweet sorghum (*Sorghum bicolor*) [157], cowpea [74], and pepper [158] (Table 4), and their respective whole genome assemblies can now be exploited to determine the genes conferring the resistance. For example, in cucumber, virus-induced gene silencing and qPCR identified two candidate genes—EVM0025394 and EVM0006042—within a QTL on chromosome 3 [96], offering targets for functional validation in susceptible genotypes.

**Table 4:** Details of major studies performed to identify quantitative trait loci (QTLs) for RKN (*Meloidogyne* spp.)-related traits.

Crop	Population Type	No. of Lines Used	No. of Major QTLs Identified	Location of Identified QTLs	Reference
Cotton (*Gossypium* spp.)	RIL	138	4	Chr 3, 4, 11, 17	[146]
	(M120 × Pima S-6) F2	245	1	Chr 14	[159]
Peanut (*Arachis hypogaea*)	RIL	93	4	LG02, 04,09	[155]
Sorghum (*Sorghum bicolor*)	(PI 144,134 × Collier) F2	249	1	Chr 5	[160]
Cowpea (*Vigna unguiculata*)	RIL, F2:3	389	1	VuLG11	[161]
	RIL	264	2	Vu01 and Vu04	[74]
Carrot (*Daucus carota*)	Two F2 mapping populations, (Br1091 × HM1) and (SFF × HM2), and one segregating HM3 population	-	5	Chr 1,2,4,8,9	[152]
Pepper (*Capsicum annuum*)	(YW × DLL) F2:3	130	4	Chr 1,9	[160]
Sweet Potato	Tanzania × Beauregard	240	9 (7 in Tanzania and 2 in beauregard)	T01.01, T05.26, T07.37, T07.38, T07.39, T07.41, T08.46	[162]
	TB population, F1	244	1	IbLG07	[154]
Soybean (*Glycine max*)	RIL (Magellan × PI 567305)	242	2	Chr 10, 13	[163]

The advent of NGS has enabled the development of high-density linkage maps, increasing the resolution of QTL mapping. However, traditional capture limited allelic diversity. The adoption of multi-parent populations, such as a nested association mapping (NAM) population or a multi-parent advanced generation intercross (MAGIC) population, has improved the power to detect QTLs and understand allelic interactions [164].

Despite technological advances, many RKN-related QTLs remain broad and inconsistently mapped, complicating candidate gene identification and incorporation into breeding programs. Meta-QTL analysis offers a solution by integrating QTL data across studies to identify robust and reliable genomic regions [165] and candidate gene identification [166]. In polyploid crops, genomic mapping is quite challenging for several reasons. Firstly, there are difficulties in connecting genotype to phenotype when the exact number of chromosome sets (ploidy), gene copies, and alleles is unknown. Second, it is unpredictable how the chromosomes pair during meiosis, which adds to the complexity. Third, many polyploid crops reproduce through outcrossing, resulting in highly heterozygous genomes. Finally, polyploid crops have more chromosomes in each homologous set, increasing the number of potential gene combinations and making trait inheritance analysis challenging. All of these characteristics have an impact on the estimation of genetic parameters, including the recombination fraction and gene effects of QTLs on phenotypes, which require additional research and consideration in the framework of polyploid QTL mapping [167]. In polyploid crops such as cotton and sweet potato, larger population sizes and higher dosage markers enhance the statistical power and resolution [162], and facilitate the construction of integrated maps, enabling accurate tracking of homoeologous loci. Additionally, the abundance of markers makes it possible to capture the whole range of recombination events [168].

### 6.3. GWAS for RKN-Related Traits

GWAS leverage natural variation across diverse germplasm to identify loci associated with traits [169], including RKN resistance. Unlike QTL mapping, GWAS utilizes historical recombination events for finer resolution and broader allelic discovery [170,171].

GWAS for RKN resistance has been performed in *Arabidopsis*, rice, soybean, sweetpotato, and common bean (*Phaseolus vugaris*) (Table 5). Publicly available germplasm resources, such as SoySNP50K (20,087 soybean accessions with 42,509), Cottongen (19,827 cotton accessions with 459,825 SNPs), and iSelect (170 elite European barley cultivars), have facilitated these efforts [172,173,174,175]. For instance, in rice, GWAS identified 11 QTLs and several lectin domain-containing, and *Mla* homologous genes on chromosome 11, previously linked to pathogen resistance in another crop [176]. A study on Indian rice by Hada, et al. [177] identified 40 RKN-resistant accessions and 17 novel SNPs linked to galling and egg mass traits. The study also highlighted resistance genes, including Cf2/Cf5-encoding genes, several TFs belonging to diverse families (i.e., MYB, bZIP, ARF, WRKY, and SCARECROW), and NBS-LRR genes. In soybean, a region on chromosome 13 harboring TIR-NB-LRR genes was associated with resistance to *M. javanica* [178].

Similarly, following a genotyping-by-sequencing (GBS)-enabled GWAS [184,185] approach, three significant SNPs out of 46,196 involved in RKN resistance in 20 soybean chromosomes were identified [180]. All three important GWAS loci are located close to QTL hotspots previously identified through mapping studies on chromosome 10. GWAS helped improve the loci’s confidence level and reduced the confidence interval. However, contrary to meta-QTL attempts, the data from many GWAS studies have not yet been assembled and used for meta-GWAS analysis. One of the causes of this might be the dearth of RKN-related GWAS research compared to QTL mapping. In the near future, GWAS efforts will undoubtedly increase owing to the expanding public resources for genotyping and whole-genome re-sequenced crop lines.

The majority of GWAS in various crops are conducted using the traditional GWAS approach, which uses logistic or linear regression analysis and is conducted separately for each SNP. This results in the identification of the genomic regions from an extensive array of SNPs that are linked to the specific trait or phenotype of interest. Subsequently, the *p*-values are used to rank the SNPs, and those with *p*-values less than <0.05 are selected [186]. However, there are drawbacks to using the traditional method because it assumes that each SNP functions independently, raising doubts about the reliability of the discovered trait–locus relationships. Additionally, it can occasionally result in false-positive SNP identifications because of linkage disequilibrium and a Gaussian distribution of phenotype [187]. Owing to these limitations, approaches that consider both population-relatedness–false correlations and epistatic interactions have been put forward [188]. However, the large number of pairwise tests that must be conducted in a single GWAS study remains a shortcoming when using the linear model [189]. Recent machine learning (ML)- based GWAS approaches, such as Random Forest (RF) and Support Vector Machine (SVM) algorithms, address these challenges by accounting for marker interactions and epistasis. However, the ML-based GWAS is not as powerful for simultaneously accounting for a wide range of interrelated physiological and biological processes and mechanisms that make up the desired phenotype [187]. ML-based pipelines have identified novel RKN resistance loci on soybean chromosomes 10 and 11, improving prediction accuracy and minimizing overfitting. This new ML-based approach of GWAS could be beneficial to improve the ability of breeding programs to identify resistant genotypes through marker-assisted selection and/or genomic prediction early in the breeding pipeline and could be translated to other crops to identify the RKN-resistant chromosomal regions [181].

### 6.4. GS: A Promising Tool to Improve RKN Resistance

GS is a derivative of MAS, which uses genotypic data to predict how a complex trait will behave phenotypically. Unlike MAS, which relies on a small number of linked markers, GS leverages dense marker coverage throughout the whole genome, capturing small-effect QTLs, often missed by MAS [190]. In GS, two population sets known as the Training set (TS) and Validation set (VS) are used (Figure 2). The TS population is both genotyped and phenotyped for the trait of interest and to estimate the marker effect, while the VS population is genotyped only. Genomic estimated breeding values (GEBVs), derived from marker effects in the TS, are used to predict phenotypes in the VS. Using the cross-validation method, which excludes a portion of the TS during model training so that the GEBVs of the VS lines may be compared to their phenotypic values, the prediction accuracy of the phenotypes is estimated. This prediction accuracy is referred to as the “predictive ability”, which is a correlation between the GEBVs and the phenotypic values obtained from the VS. The prediction ability is the marker of prediction accuracy, which is defined as the predictive ability divided by the square root of heritability [191]. Techniques like QTL mapping and GWAS that rely on high-throughput genotyping, phenotyping, and a large number of genotypes are usually integrated with GS approaches.

Utilizing genomic predictions to choose parents with complementary genetic information that could be combined to produce superior offspring, GS can also be employed even sooner in a breeding program. This method, known as genomic mating or genomic selection of parents [192], screens parental combinations and chooses which crossings to do and how many field resources to allot to each progeny using genetic predictions. A genetic map and genotypic data from possible parents are used to simulate a segregating population. Afterward, phenotypic values are predicted for each progeny line, and population statistics are computed for each cross [193]. Crosses that exhibit high progeny performance across all target traits could then be created. Due to higher progeny variation or a lower within-progeny correlation between negatively correlated characteristics, crossings with more transgressive segregants will subsequently yield the most genetic gain when more breeding resources are allocated to them.

In cowpea, GS has been successfully applied to analyzing grain nutrition traits in a MAGIC population [194,195], utilizing a broad suite of available cowpea genomic resources [196]. The cowpea MAGIC population has some of the eight parents carrying RKN resistance, and GS could be tested for selection in resistance breeding in this system. Therefore, genomic selection and genomic mating represent promising tools for accelerating the development of cultivars with improved RKN resistance.

### 6.5. Taking Advantage of Whole-Genome Resequencing to Track Down RKN Resistance Traits

NGS platforms provide a valuable opportunity for high-throughput identification and genotyping of map populations by whole-genome resequencing (WGR) at low coverage [197]. The approach is inexpensive and takes advantage of the availability of high-quality reference genomes for economically important crops to identify domestication sites, perform selective sweeps, increase genetic diversity, improve population structure, analyze data on genetic gain and loss during evolution, and explore potential genotypes for crop improvement [198]. The allele mining approach using resequencing information on diverse genotypes can facilitate the study of candidate gene(s)/QTL, identify haplotype blocks associated with specific phenotypes, and develop allele-specific markers for breeding programs [199]. To date, this approach has been implemented in several crops, including soybean [61], and has facilitated the analysis of genome resequencing data associated with selection, domestication, CNVs, genes responsible for qualitative (such as specific enzyme or protein and fatty acid biosynthesis) and quantitative traits (such as fruit shape and color, plant shape), and QTLs for several agronomic traits (flowering, plant height, primary and secondary branches per plants) [61,200,201,202,203].

Only a few studies have implemented this approach to identify QTL and candidate genes for RKN resistance. For instance, Xu, et al. [204] generated 246 recombinant inbred lines (RIL) of soybean derived from the cross between the RKN-resistant (PI 438489B) and the RKN-susceptible (Magellan) parent lines to test for RKN resistance. The generated lines were sequenced at an average of 0.19× depth to generate a bin-map. Using these data, a subsequent linkage map was developed with bins serving as markers, enabling more accurate mapping of QTLs related to RKN resistance and the genes underlying these QTLs, thus surpassing the time-consuming and laborious fine-mapping process. The study led to the identification of the three major QTLs and two significant genes for RKN resistance, namely *Glyma10g02160* and *Glyma10g02150*, which encode a pectin methylesterase inhibitor—pectin methylesterase and a pectin methyltransferase inhibitor, respectively. Similarly, a linkage bin-map for the genotyping of an RIL population, produced by crossing the cucumber cultivar ‘Beijingjietou’ CC3 with RKN-resistant introgression line IL-01, was successfully created in cucumber utilizing a parent-independent approach. This study led to the identification of three genomic regions containing RKN resistance QTLs harboring 37 genes with nonsynonymous SNPs. Four of those genes, encoding a leucine-rich repeat receptor protein kinase-like protein (*Csa5M610420*), a leucine-rich repeat (LRR) family protein (*Csa5M608240*), pathogenesis-related 5-like receptor kinase (PR5K, *Csa5M610370*), and a programmed cell death protein (PCD, *Csa5M623410*), were considered as candidate genes for RKN resistance [205]. The findings of these studies may help identify novel nematode-resistance genes, QTL, and haplotypes for breeding strategies and trace the evolution of nematode resistance from wild races to domesticated cultivars. However, there is a need to validate these candidate genes and test their functionality in providing resistance to RKN.

The resources generated through whole-genome sequencing and resequencing must be better explored to dissect the QTL hotspots that provide RKN resistance. In a study on sweetpotato, a total of 46,982 SNPs were found throughout the genome using double-digested restriction site-association DNA sequencing (ddRAD-seq). Using these data, a novel approach to GWAS was used that uses multiple-dose markers, and genetic mapping was carried out to calculate the allele dosage probability for each SNP. Upon the development of markers based on the DNA sequence, the SNPs associated with RKN resistance were identified on chromosomes 3 and 7. This method effectively identified genomic regions of agronomically significant traits, specifically resistance to RKN in the polyploid crop sweetpotato. It may also be useful in identifying the same traits in other polyploid crops, including cotton, canola (*Brassica napus*), and wheat, and thus may help to direct future genetic mapping in these crops for RKN resistance [183].

## 7. Conclusions and Future Directions

Plant parasitic nematodes are responsible for a yearly yield loss in the U.S. of more than $1 billion. Host resistance, in combination with chemical treatment or cultural tactics, has been the most effective strategy for controlling RKN. Unfortunately, cultivars resistant to RKN that are available to farmers are limited. Therefore, it is crucial to create new cultivars that are resistant to nematode infestations in order to limit yield losses. However, there is still a dearth of diversity for RKN resistance in the present cultivars. It is vital to emphasize the expansion of our understanding of the infection mechanisms and the plant defense responses, as well as finding new resistance genes and QTLs that can be used to develop effective approaches for RKN control. Therefore, continual germplasm screening to identify new varieties with novel gene pools becomes essential for improving RKN resistance. Identification of these RKN resistance sources will prove beneficial for the development of novel germplasm populations and varieties [206].

Newly identified resistance genes and QTLs can be incorporated or pyramided into the new crop varieties using conventional breeding or genome engineering approaches, hence facilitating improvement in the RKN resistance availability. For example, the expression of R genes like *Mi-1*, *NTR1*, *RBM3*, *NPR1*, and *AOC* could be manipulated in susceptible genotype to achieve high RKN resistance, as demonstrated in earlier studies (Figure 2). Moreover, only a few S genes, such as *LMM1*, *MS*, *HIPP27*, and *AAP6*, previously identified through genome editing approaches, have been tested for RKN resistance in a few species. These genes can also be altered in susceptible genotypes of economically important crops to produce RKN resistance. Similarly, resistance-providing genes such as *LOX* and *COI-JAZ*, which are upregulated during RKN infection in various crops and have been identified through transcriptomic efforts, could be considered for further validation and to make newly resistant crops economically significant. Importantly, more research to explore the role of microRNAs (miRNAs) in plant–RKN interactions can help expand and strengthen current management strategies, as just a few miRNAs have been identified to control RKN resistance via plant hormone signaling [207].

In the past few decades, significant improvements have been made in the area of genomics and genetic technologies, along with bioinformatic tools that are useful for breeding programs. The advancements in these areas resulted in the birth of next-generation breeding strategies. Currently, new genetic sources, publicly available reference genomes, and WGR information enable breeders to identify new genes and QTLs for desired traits more quickly. This discovery process is used to study the evolutionary history of these genes to exploit the different positive alleles from both wild and exotic germplasm and, hence, serves pre-breeding programs by identifying the haplotype of desired alleles from different germplasm and wild crop lines. In the near future, advancements in sequencing technologies, bioinformatics tools, and high-throughput phenotyping will boost current breeding schemes for better characterization, more effective allele mining, and crop breeding. However, there will be significant challenges in linking all available genomic information with efficient and robust phenotypes for a wide range of desired traits [199]. In addition, beyond advances in the understanding of the genetic resistance loci, progress has also been made in the field of genomics and genetics of nematode–plant interactions [25]. These studies will serve as a platform for the discovery of novel host plant resistance genes and nematode effectors that could then be used in combination with molecular genetic engineering techniques like transgene overexpression, gene editing, and RNA interference (RNAi) to confer nematode resistance.

## Figures and Tables

**Table 1:** Representation of effectiveness in omics vs. conventional breeding [57,58].

Attributes	Conventional Breeding	Omics-Based Breeding
Example of approach	GS, MAS, GWAS, QTL mapping	Transcriptomics, genomics, metabolomics, lipidomics, proteomics
Labor intensity	Highly labor extensive	Moderately labor extensive
Cost	Low to moderate	Moderate to low
Precision	Low, since it is based primarily on phenotype	More accurate because it is based on genotype
Time requirement	Dependent on crop cycle; 8–12 years to release an improved variety	From weeks to a few months to generate data; candidate gene(s) responsible for the trait(s) is then identified and assessed through overexpression, silencing, and gene editing approaches; generation of transgenic crops can take from a few months to 1–2 years based on genotype
Regulation	Flexible regulation and release of germplasm; dependent on country regulations	Strict regulatory frame depending on the country, e.g., under regulation and approval from USDA, APHIS, and FDA in the USA
Variability	Highly variable, as it is created by hybridization; low number of replicates	Low variability; most approaches are high-throughput and allow a high number of replicates
Accessibility	Widely practiced as no special equipment is required	Requires sophisticated instrumentations and expertise
Reliability	Less reliable because it is based on phenotype and breeder’s subjective analysis	Highly reliable, though dependent on genotype
Other	Provide potential benefits to consumers, farmers, and the environment	Provide potential benefits to consumers, farmers, and the environment Provide acknowledgement and resources for marker-assisted selection and GS, MAS, GWAS, and QTL mapping Provide acknowledgement that helps develop more effective and safer strategies/technologies to control pests and diseases, and allows conceptual advances in plant biology/physiology and other related fields

**Table 2:** Details of transcriptomics studies on RKN (*Meloidogyne* spp.) infection.

Crop	Platform	Total No. of DEGs		Key Findings *	Reference
		Susceptible Line	Resistant Line		
Alfalfa (*Medicago sativa*)	Illumina Hi-Seq 2000	1143	319	R genes, signaling pathways, oxidative stress, chemical stimulus, antioxidant activity, oxidoreductase and peroxidase activity	[66]
Cowpea (*Vigna unguiculata*)	Affymetrix GeneChip expression array	1060	552	Genes related to ROS, toxins, and defense	[76]
Eggplant (*Solanum melongena*)	Illumina Hi-Seq 4000	8148	4761	Genes related to cell wall biogenesis/organization, stimulus, hormone, plant hormone signal Transduction, and plant–pathogen interaction	[79]
Pepper (*Capsicum annuum*)	Illumina Hi-Seq	2057	1217	Genes located on chromosome 9 (NBS-LRR resistance gene, genes belonging to transcription factors or kinases)	[80]
Tomato (*Solanum lycopersicum*)	Illumina Hi-Seq 2000	1827	25	Cell wall structure, development, primary and secondary metabolism, defense signaling pathway, hormone- mediated defense response	[16]
Sweetpotato (*Ipomoea batatas*)	Illumina Hi-Seq 2000	881	929	Genes related to hormone signaling-related transcription factors, PR genes	[81]
Tobaco (*Nicotiana tabacum*)	Illumina Hi-Seq 2000	545	4354	Genes related to cell wall modification, toxic compound synthesis, ROS, salicylic acid signal transduction and metabolites	[82]
	Illumina Hi-Seq 2000	545	2623	Auxin-related proteins, cell wall modifying proteins, ROS	[83]
Soybean (*Glycine max*)	Illumina Hi-Seq 4000	5842	7041	Genes related to mTOR, OI3K-Akt, thermogenesis, relaxin and phenylpropanoid pathway	[84]
Peach (*Prunus kansuensis*)	Illumina Hi-Seq 2000	1476	2107	Genes related to phytohormone metabolism	[85]
Cotton (*Gossypium hirsutum*)	Illumina Hi-Seq 300	1355	1250	Cell wall organization, defense response, phytohormones, protein serine/threonine kinase activity	[86]
	Illumina Hi-Seq 2500	8247	1093	Phytohormone signaling (particularly salicylic and jasmonic acid), cell surface-related receptors	[70]

* differentially expressed genes and transcription factors.

**Table 3:** Molecular marker resources available for RKN (*Meloidogyne* spp.) studies.

Crop	Nematode Species	Marker Type	Resistance Gene	References
Tomato (*Solanum lycopersicum*)	*M. incognita*, *M. Javanica*	CAPS	*Mi-1*	[133]
	*M. incognita*, *M. arenaria*, *M. javanica*	RAPD	*Mi1.1*, *Mi1.2*	[134]
	*M. incognita*	RAPD, RFLP	*Mi 3*	[135]
Cucumber (*Cucumis metuliferus*)	*M. Javanica*	AFLP and SRAP	*mj*	[136]
Turmeric (*Curcuma longa*)	*M. incognita*	ISSR	*-*	[137]
Cotton (*Gossypium hirsutum*)	*M. incognita*	SSR	*qMi-C14*	[138]
	*M. incognita*	AFLP and derived CAPS	*rkn1*	[130]
	*M. incognita*	SSR	*-*	[132]
Peanut (*Arachis hypogaea*)	*M. arenaria*	RFLP	*-*	[139]
	*M. arenaria*	CAPS, SSR, AFLP	*Rma*	[140]
Soybean (*Glycine max*)	*M. incognita*	SSR	*Rmi*	[141]
Mulberry (*Morus* spp.)	*M. incognita*	SSR	*-*	[142]
Carrot (*Daucus carota*)	*M. javanica*	RAPD and STS	*Mj-1*	[143]
	*M. incognita*	SSR	*Mj-1*	[144]
Eggplant (*Solanum melongena*)	*M. javanica*	RT-PCR	*Mi-1.2*	[95]
Pepper (*Capsicum annuum*)	*M. incognita*, *M. arenaria*, *M. javanica*	RAPD, RFLP	*Me_3_* and *Me_4_*	[145]

**Table 5:** Details of major genome-wide association loci governing RKN (*Meloidogyne* spp.)-related traits.

Crop	Platform/Technique	No. of Genotypes	No. of Loci Tested	Reference
*Arabidopsis thaliana*	Association mapping	340	214,051	[179]
Indian wild rice (*Oryza* spp.)	50K “OsSNPnks” genic Affymetrix chip	272	50,051	[177]
Asian Rice (*Oryza sativa*)	44K Affymetrix SNP chip	332	44,100	[176]
Soybean (*Glycine max*)	GBS	317	44,992	[178]
	GBS	193	46,196	[180]
	BARCSoySNP6K BeadChip	717	4974	[181]
Common bean (*Phaseolus vulgaris*)	Association mapping	180	10,362	[182]
Sweetpotato (*Ipomoea batatas*)	GBS	107	46,982	[183]

## Abbreviations

The following abbreviations are used in this manuscript:

AFLPs	Amplified fragment length polymorphisms
*AOC*	*Allene oxide cyclase*
ARF	Auxin response factors
*AtAAP6*	*Arabidopsis thaliana Amino Acid Permease 6*
*AtHIPP27*	*Arabidopsis thaliana Heavy Metal-Associated Isoprenylated Plant Protein 27*
BZIP	Basic leucine zipper
BZIP60	Basic leucine zipper 60
CAPS	Cleaved amplified polymorphic sequence
CNVs	Copy number variants
*COI-JAZ*	*Coronatine insensitive 1-jasmonate zim-domain*
CRISPR/Cas9	Clustered regularly interspaced short palindromic repeats/CRISPR-associated protein 9
*CsMS*	*Cucumis sativus malate synthase*
DAMPs	Damage-associated molecular pattern
ddRAD-seq	Double-digested restriction site-association DNA sequencing
DEGs	Differentially expressed genes
*DUF538*	*Domain of unknown function538*
EMS	Ethyl methanesulfonate
ET	Ethylene
ETI	Effector-triggered immunity
GBS	Genotyping-by-sequencing
GEBVs	Genomic estimated breeding values
*GhDIR4*	*Gossypium hirsutum dirigent protein4*
*GhPRXIIB*	*Gossypium hirsutum peroxiredoxin 11B*
*GmLMM1*	*Glycine max lipid metabolism modulator 1*
GS	Genomic selection
GT-3A	Trihelix transcription factor GT-3a
GWAS	Genome-wide association mapping
JA	Jasmonic acid
J2	Second stage RKN juveniles
*MAPK*	*Mitogen-activated protein kinase*
MAGIC	Multi-parent advanced generation intercross
MAS	Marker-assisted selection
MeJA	Methyl jasmonate
MiMsp40	*M. incognita* esophageal gland cell secretory protrein40
ML	Machine learning
MiPFN3	Meloidogyne incognita profilin 3
ML	Machine learning
MPKs	Mitogen-activated protein kinases
MYB	Myeloblastosis
NAM	Nested association mapping
NBS-LRR	Nucleotide-binding site—leucine-rich repeat
NGS	Next-generation sequencing
NLR	Nucleotide-binding leucine-rich repeat
NPR1	*Non-expressor of pathogenesis-related genes-1*
*NtRK1*	*Nicotiana tabacum Receptor Kinase 1*
*OsHPP04*	*Oryza sativa copper metallochaperone heavy metal-associated plant protein 04*
*OsLOX7*	*Oryza sativa lipoxygenase 7*
ONT	Oxford nanopore technologies
*OsThion2*	*Oryza sativa thionin2*
PacBio	Pacific Biosciences
*PAL*	*Phenylalanine ammonia-lyase*
PAMPs	Pathogen-associated molecular patterns
PBL	Plant bap-like
PCD	Programmed cell death protein
*PI-II*	*Proteinase Inhibitor II*
*PLA_2_*	*Phospholipase A_2_*
PR	Pathogenesis-related
PRR	Pattern recognition receptors
PR5K	Pathogenesis-related 5-like receptor kinase
PTI	Pattern-triggered immunity
QTL	Quantitative trait locus
RALFs	Rapid alkalinization factors
RALPs	Restriction amplified length polymorphisms
RAPD	Random amplified polymorphic DNA
*RBM2*	*RNA-binding motif protein 2*
*RBM3*	*RNA-binding motif protein 3*
RF	Random forest
RIL	Recombinant inbred lines
ROS	Reactive oxygen species
RKN	Root-knot nematode
RT-PCR	Reverse-transcription polymerase chain reaction
RNAi	RNA interference
SA	Salicylic acid
SCAR	Sequence characterized amplified region
SCL	SCARECROW-like
*SlARF8A*	*Solanum lycopersicum auxin response factor 8A*
SNPs	Single nucleotide polymorphisms
*Spr2*	*SPIRAL2*
SSR	Single sequence repeats
SVM	Support vector machine
TFs	Transcription factors
TIR-NBS-LRR	Toll/interleukin-1 receptor—nucleotide-binding site–leucine-rich repeat
TM-1	Texas marker-1
*TUB-1*	*Tubilin-1*
TS	Training set
VQ	Valine–glutamine
VS	Validation set
WGCNA	Weighted gene co-expression network analysis
WGR	Whole-genome resequencing
WMJ	Wild Mexican Jones
WRKY	Tryptophan–arginine–lysine–tyrosine (WRKY)
